# Replicative Homeostasis: A fundamental mechanism mediating selective viral replication and escape mutation

**DOI:** 10.1186/1743-422X-2-10

**Published:** 2005-02-11

**Authors:** Richard Sallie

**Affiliations:** 1 Suite 35, 95 Monash Avenue, Nedlands, Western Australia, Australia

## Abstract

Hepatitis C (HCV), hepatitis B (HBV), the human immunodeficiency viruses (HIV), and other viruses that replicate via RNA intermediaries, cause an enormous burden of disease and premature death worldwide. These viruses circulate within infected hosts as vast populations of closely related, but genetically diverse, molecules known as "quasispecies". The mechanism(s) by which this extreme genetic and antigenic diversity is stably maintained are unclear, but are fundamental to understanding viral persistence and pathobiology. The persistence of HCV, an RNA virus, is especially problematic and HCV stability, maintained despite rapid genomic mutation, is highly paradoxical. This paper presents the hypothesis, and evidence, that viruses capable of persistent infection autoregulate replication and the likely mechanism mediating autoregulation – Replicative Homeostasis – is described. Replicative homeostasis causes formation of stable, but highly reactive, equilibria that drive quasispecies expansion and generates escape mutation. Replicative homeostasis explains both viral kinetics and the enigma of RNA quasispecies stability and provides a rational, mechanistic basis for all observed viral behaviours and host responses. More importantly, this paradigm has specific therapeutic implication and defines, precisely, new approaches to antiviral therapy. Replicative homeostasis may also modulate cellular gene expression.

## Background

### 1. Disease burden

Hepatitis C (HCV), HBV and HIV are major causes of premature death and morbidity globally. These infections are frequently life-long; Hepatitis viruses may result in progressive injury to the liver and cirrhosis, and death from liver failure, or hepatocellular carcinoma, while HIV causes progressive immune depletion and death from the acquired immunodeficiency syndrome (AIDS). Together, these infections cause millions of premature deaths annually, predominantly in "developing" countries. Other viruses replicating via RNA intermediaries cause similar morbidity among domestic and wild animal populations. While education, public health measures and vaccination (for HBV) have resulted in significant progress in disease control, therapy of established viral infection remains unsatisfactory.

### 2. Viral replication

RNA viruses and retroviruses replicate, at least in part, by RNA polymerases (RNA_pol_), enzymes that lack either fidelity or proofreading function [76]. During replication of hepatitis C HCV or HIV each new genome differs from the parental template by up to ten nucleotides [61] due to RNA_pol _infidelity that introduces errors at ~1 × 10^-5 ^mutations / base RNA synthesised.

**Figure 1:**
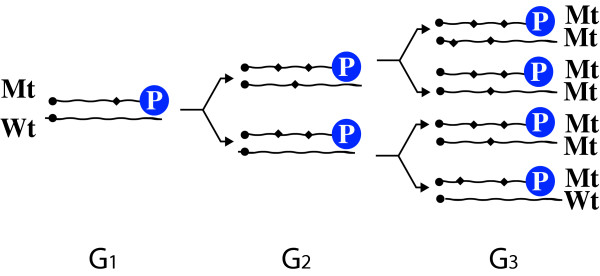
**Effect of RNApol fidelity on replication**. Each replication cycle may produce either wild-type (Wt) or variant (Mt) copies of parental template in a ratio determined by polymerase fidelity. If HCV RNA_pol _M_u _is 10^-5 ^mutations per base RNA synthesized, Mt:Wt ratio at G_1 _is ~9:1, by G_3 _unmutated parental genome is 6.8 × 10^-4^of total virus population, and by G_20 _7.5 × 10^-22^

Viruses replicate by copying antigenomic intermediate templates and hence obey exponential growth kinetics, such that [RNA]_t _= [RNA]_(t-1)_e^k^, where [RNA]_t _is virus concentration at time (t) and k a growth constant. However, because of RNA_pol _infidelity, wild-type (wt) virus will accumulate at [RNA_wt_]_t _= [RNA_wt_]_(t-1)_•(1-ρ)•K_1 _and variant forms (mt) at [RNA_mt_]_t _≈ ([RNA_wt_]_(t-1)_•ρ + [RNA_mt_]_(t-1)_)•K_1_, where ρ is the probability of mutation during replication and K_1 _= e^k^. Therefore, while wild-type virus predominates early, replication (and intracellular accumulation) of variant virus and viral proteins will accelerate (in a ratio of ([RNA_wt_]_(t-1)_•ρ + [RNA_mt_]_(t-1)_)/ [RNA_wt_]_(t-1)_•(1-ρ) compared to wild type) and variant viral RNAs will rapidly predominate (Figure 1). Mutations progressively accumulate in RNA viruses [17] and ultimately variant RNAs and proteins, if variant RNAs are translated, will become dominant. It is also likely some variant viral proteins will resist cellular trafficking, further accelerating the intracellular accumulation of variant forms relative to wild type.

## The paradox of quasispecies stability

Two fundamental problems critical to understanding RNA virus quasispecies biology arise because of RNA polymerase infidelity and the mode of viral replication:

### 1: Replication kinetics

**Figure 2:**
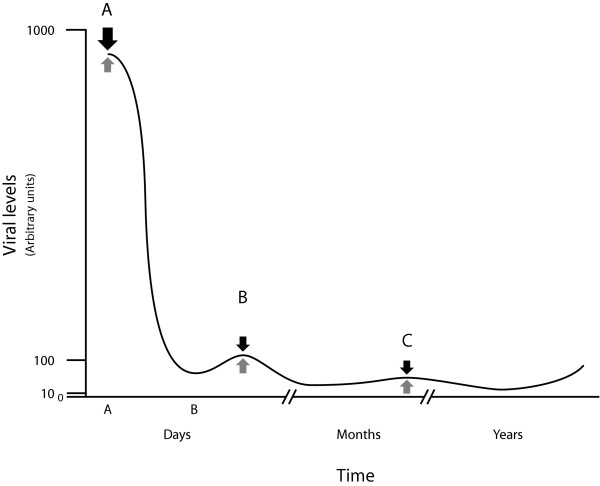
**Viral kinetic paradox**. Viral replication kinetics (—). If host factors (I_c_, black arrows) reduce viral replication acutely (point A), then they must exceed viral forces (V_e_, grey arrows). At equilibrium (e.g. points B or C) host forces must balance viral forces; I_c _must therefore fall by a factor of 10^2–3 ^from A.

Hepatitis C, HIV, and HBV and other viruses, have broadly similar kinetics (Figure 2); initial high level viral replication that rapidly declines to relatively constant low-level viraemia [11,12], typically 2–3 logs lower than at peak, for prolonged periods, a kinetic profile attributed to "immune control" [12]. However, immune control is a conceptually problematic explanation for the initial decline in viral load; For example; why would potent host responses (of whatever type; humoral, cell mediated or intracellular immunity, or any combination thereof), having reduced viral load and antigenic diversity by a factor of 10^2–3 ^within days, falter once less than 1% of virus remains?

#### Formally

1. Assume immune mechanisms reduce initial viral replication.

2. Let I_c(t) _represent the immune forces favouring viral clearance and V_e(t) _viral forces promoting quasispecies expansion pressures at time (t).

3. Assume immune pressures I_c _required to clear virus are proportional to viral concentration [V], that is; V_e _∝ [V] (or V_e _= k_e _[V] where k_e _is some constant), so that I_c _required to clear one viral particle I_c(1) _is less than that I_c _required to clear 10 viral particles Ic_(10)_.

4. At equilibrium (e.g. time points B or C, Figure. 2) immune clearance pressures approximate viral antigenic expansion pressures: I_c(b or c) _≈ V_e(b or c)_.     Eq.1

5. If I_c _causes the reduced viral load seen between time A and time B or C, [V_e(a)_] ⇒ [V_e(b or c)_], then immune clearance pressures must exceed viral expansion pressures at that time i.e. I_c(a) _> V_e(a)_.     Eq.2

6. As viral antigenic expansion pressures at time A exceed those at time (B or C) by 10^2–3 ^[V_(a)_] ≈ [V_(b or c)_]• 10^2–3^, and I_c(b or c)_= V_e(b or c) _then immune clearance pressures at time A exceed those at time (B or C) by10^2–3 ^I_c(a) _>I_c(b or c)_• 10^2–3^. That is, immune pressures fall by 10^2–3 ^between time A and B or C, (Figure. 2).

#### Prompting

i) Why, and by what mechanism, would immune forces, or any other host defense mechanisms, fall by 10^2–3 ^over days between time A and B or C?

There is, of course, no evidence immune pressures fall, and very considerable evidence both antibody and adaptive T cell responses are increasing when viral replication is falling [5,12]. These facts are irreconcilable with the notion that immune or other any host mechanisms control initial viral replication and strongly suggest immune or any other host mechanism(s) are not the primary reason viral load falls initially. Further, as down-regulation of viral replication frequently occurs prior to development of neutralising antibody, in the absence of any demonstrable antiviral antibody, or T-cell responses [25,41], and without lysis of infected cells [25], it is difficult to argue, with any conviction, that either humoral or cellular immune responses primarily cause reduced viral replication. Evidence that prior HCV infection does not confer protective immunity against either heterologous HCV infection in chimpanzee [22]or either homotypic [33] or heterotypic [32] human reinfection further undermines the paradigm of "immune control". Inhibition of immune or other host mechanisms is an untenable explanation of this massive apparent fall in immune clearance pressures; if occurred to any degree, an increase, rather than the observed decrease, in viremia would result. In the absence of a rational host mechanism consistent with observed viral kinetic data, the ineluctable conclusion is that non-host (i.e. viral) mechanisms (i.e. viral auto regulation) must be operative.

Chronic viral persistence raises other issues; At steady state (e.g. points B or C, Figure. 2), the rate of HIV and HCV production is estimated at 10^10 ^molecules / day [11,29,52,57] while HBV production may be 10^11 ^molecules/day resulting in an average viral load of 10^10 ^molecules/person [52,57]. However, during peak replication virus production may 10^2–3 ^times the basal rate [11,12], indicating enormous reserve replicative capacity. As basal viral replication is clearly sufficient for long-term stability, and kinetic analysis suggests viral, rather than host, factors control viral replication, the following questions are posed: When challenged, how do viruses "sense" the threat and by what mechanism do they modulate replication in response?

### Problem 2: Mutation rate

**Figure 3:**
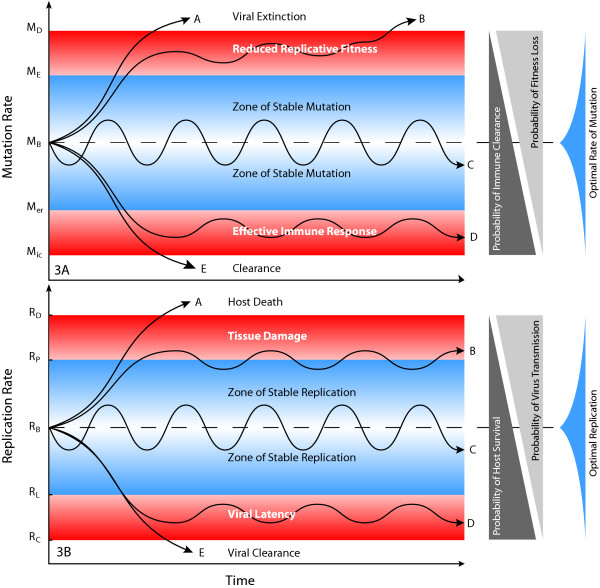
**a. Constraints on viral mutation**. Inadequate polymerase fidelity will cause loss of sequence information and quasispcies extinction (A, B), while inadequate viral mutation will result in immune recognition and viral clearance (D,E). Viral persistence requires polymerase fidelity responsive to the host environment (C). **3b. Constraints on viral replication**. Overly rapid replication will cause cell lysis, tissue injury and premature host death (A,B), while inadequate replication will result viral latency or clearance (D,E). Viral persistence with optimal evolutionary stability requires a polymerase responsive to the host environment (C).

The stability of RNA viral quasispecies poses a major problem: During viral replication the copied genome may either identical to or a variant of parental template (Figure. 1). The probability (ρ) of a mutation occurring during replication is a function of polymerase fidelity; During one replication cycle ρ = (1-(1-M_μ_)^n^), where (M_μ_) is mutation rate and (n) genome size. Hepatitis C (a ~9200 bp RNA virus) RNA_pol _introduces mutation at 10^-5 ^substitutions/base, ρ≈0.912. However, for multiple (θ) replications cycles, ρ = (1-(1-M_μ_)^n^)^θ^. After 20 replication cycles, occurring in <7 days in most patients [52,57], the probability of any original genome remaining un-mutated is ρ_o_≈7.5 × 10^-22^, meaning effective loss of sequence information, an outcome that should cause quasispecies extinction [16]. Persistence of stable RNA viral quasispecies is, therefore, highly paradoxical [18]. This "theoretical impossibility" of RNA quasispecies stability suggests either a) the consistently reported rates of RNA_pol _infidelity are incorrect (which, even if true, would only delay quasispecies extinction; if M_μ _= 10^-10^, ρ_o _<10^-40 ^within 100 days etc.) or b) that innate viral mechanism(s) control RNA_pol _fidelity and mediate selective replication of consensus sequence genomes. Thus, rates of viral mutation are tightly constrained by the necessity to retain sequence information. On the other hand, overly faithful template replication will restrict antigenic diversity, rendering virus susceptible to immune destruction and unresponsive to ongoing cellular changes. The necessity to retain sequence information by adequate replicative fidelity, and the later requirements (in terms of replicase ⇒ RNA_pol _evolution) of viruses to access cells via evolving cell receptors and evade host defence mechanisms, has placed constraints on replicase (RNA_pol_) function that dictate polymerase fidelity must be tightly, and dynamically, controlled (Figure 3a).

### Evolutionary constraints on viral replication

Optimal viral replication is a compromise between maximising host-to-host viral transmission at each host contact versus maximising transmission at sometime during the host's life: Uncontrolled, exponential growth, as might result from the mode of viral replication, would cause rapid cell lysis, host death and a reduced likelihood of stable host-to-host transmission, a prerequisite for viral survival on an evolutionary timescale. While maximising the probability of host-to-host transmission at each contact, high-level viral replication increases the probability of host disease, thus reducing opportunity for transmission long term. Contrariwise, adverse viral outcomes may result from inadequate viral replication causing increased clearance and reduced host-to-host transmission. Viruses that cause premature host death or that are cleared by host mechanisms before transmission to, and infection of, other hosts are biological failures that have strong Darwinian pressures acting against them. Optimal long-term viral stability, therefore, dictates viral replication rates (that is, polymerase processivity) and mutation frequency (that is, polymerase fidelity) must be closely regulated (Figure 3b).

### Hypothesis

That viruses capable of chronic persistence auto-regulate replication and mutation rates by replicative homeostasis. Replicative homeostasis results when RNA polymerase end-translation products (envelope and contiguously encoded accessory proteins) interact with RNA_pol _to alter processivity and fidelity.

### Evidence for Autoregulation

Substantial clinical and in-vitro evidence, including the kinetic paradox indicate viruses auto-regulate. During successful antiviral treatment levels of virus fall sharply [12,29,52,53,57], often becoming undetectable. However, viral replication rebounds, rapidly and precisely, to pre-treatment levels on drug withdrawal in patients [52,53,57] and in tissue culture [1]. This in-vitro data confirm replication is controlled by factors independent of either cellular or humoral immune function. Auto-regulation of HCV replication was confirmed most emphatically in patients undergoing plasmapharesis in whom 60–90% reduction in levels of virus returned to baseline, but not beyond, within 3–6 hours of plasma exchange [44]. Studies suggesting autoregulation of tobacco mosaic virus replication occurred independent of interferon effects, intrinsic interference or interference by defective virus [34] confirming this phenomenon is not confined to either animal viruses or cells. These data beg the questions: How does the replicative mechanism "choose" any particular level of replication and how does it return, so accurately, to pre-treatment levels?

### RNA polymerase control

Most cellular enzymes are under some form of kinetic control, usually by product inhibition. While simple negative-feedback product inhibition is sufficient to control enzyme reaction velocity and the rate of product synthesis, it is inadequate to ensure the functional quality of any complex molecules – including proteins – synthesised. The functionality of RNA_pol _output depends on the functionality of protein(s) translated from any RNA synthesized by RNApol. For viruses, and their polymerase, evolutionary survival – i.e. whether the polymerase, and its viral shell, avoids immune surveillance, gains access to cells, and replicates to infect other hosts – is a function of the properties that the sequence, topological variability and structural integrity of envelope proteins impart. RNA polymerase is responsive to and is influenced by accessory proteins that induce conformational changes to alter both processivity and fidelity [20,31], representing partial "proof of concept" of the mechanism postulated.

### Evolutionary stability

Evolutionary stability requires adaptability to changing environmental circumstances. For viruses, an ability to modulate replication and mutation rates dynamically in response to cellular changes is essential. Viruses intrinsically capable of adaptation to environmental changes, including variations in host density, and evolving cell receptor polymorphisms, immune and other host responses, among other variables, will enjoy a competitive advantage over viruses lacking innate responsiveness. Contrariwise, self-replicating molecules, including viruses, that lack innate adaptability, for whom replication is contingent upon a chance confluence of appropriate cellular conditions – including permissive cell receptors, absence of cell defences and so on – are highly vulnerable to extinction by both adverse environmental changes and competition for scarce intracellular resources by molecules capable of adaptation. For viruses, this adaptability requires antigenic and structural diversity be controlled and, in turn, that means the two critical RNA_pol _attributes, fidelity and processivity, be dynamically modifiable, and controllable. These linked functional requirements imply a dynamic nexus between the functional output of RNA_pol _(i.e. envelope proteins) and that polymerase.

### Homeostatic systems

Systems capable of homeostatic regulation (auto-regulation) have the following characteristics: i) an efferent arm that effects changes in response to perturbations of an equilibrium; ii) an afferent arm that measures the systems response to those changes; iii) mechanism(s) by which i) and ii) communicate. The mechanism of viral autoregulation – Replicative Homesostasis – described here requires: i) that viral envelope (Env) proteins interact with viral RNA polymerases (RNA_Pol_); ii) that these Env :RNA_Pol _interactions alter both polymerase processivity and fidelity; iii) that wild-type (consensus sequence) Env_wt _:RNA_Pol _complexes cause more rapid, less faithful RNA replication than variant (variant) Env_mt _:RNA_Pol _complexes. There is solid evidence for each requirements of replicative homeostasis.

### The Envelope-Polymerase relationship: Evidence for mechanism

A large body of literature, for many viruses, establishes an important relationship between envelope and polymerase proteins and documents that Env proteins influence both RNA_Pol _processivity and fidelity.

First, for HIV, overwhelming evidence suggests HIV polymerases properties, and those of related retroviruses – for example, simian immunodeficiency virus (SIV) and the feline immunodeficiency virus (FIV) – are influenced by Env proteins (for example, [9,15,35]. Broadly, these indicate heterologous Env proteins – when administered as live attenuated vaccines [71], adjuvant enhanced protein vaccine [83], or as recombinant Env proteins in cell culture [64] – dramatically alter viral load, and both replication and mutation rates of wild-type virus. Specific examples include data demonstrating HIV Env regions obtained from different patient isolates, when cloned into common HIV-1 backbones, conferred a spectrum of replication kinetics and cytotropisms characteristic of the original Env clone, and independent of either the clones' ability to raise antibody [51], or the replicative characteristics of the 'native' polymerase backbone [51]. Similarly, chimeric HIV-1 viruses expressing heterologous Env, again with a common polymerase backbone, have replication kinetics and cell tropism phenotypes identical to the parental Env clone [39], suggesting the Env is a critical determinant of polymerase function. Similar results obtained with SIV clones [36] strongly support conclusions drawn from feline immunodeficiency virus [37] data. Fine mapping of HIV envelope proteins identified 6 mutations within the V1-V3 loop that increased viral replication in a manner independent of nef [77], confirming other work examining HIV Env recombinants [14], and extending earlier work that demonstrated a single amino acid substitution (at position 32 of the V3 Env domain) was sufficient to change a low replication phenotype into high-replicating phenotype [13]. Finally, for HIV, co-transfection with Env variants at 10 fold excess dramatically inhibited replication of wild-type virus [75], providing direct evidence for both the interaction and differential affinity for wild-type and variant Env for polymerases. Critically, many of these observations are from in-vitro systems, indicating the effects are independent of either cellular or humoral immune influence. Many studies report the effect of Env/polymerase interactions in terms of altered viral tropisms, and did not examine changes to polymerase fidelity explicitly. However, virus replication can alter in only two ways; either there is more or less virus, or the viral genomic sequence may be changed by altered polymerase fidelity. Variant viruses expressing altered envelope proteins will have altered cell receptor affinities and hence, variable cell tropisms.

Second, for HCV, many separate observations document HCV replication and polymerase functionality is dependent on envelope proteins: i) HCV viral genotypes are defined by sequences of either envelope or polymerase regions [43,73,74] and these are necessarily acquired together – a genetic nexus implying a functional relationship. ii) Observations that a) co-infection with multiple HCV genotypes occurs less frequently than predicted by chance and b) certain HCV genotypes become progressively dominant in populations both suggest – at a population level – replicative suppression of some HCV genotypes by others [68]. These observations are supported by observations of both homotypic [33] and heterotypic HCV super-infection [32] documenting genotype-dependent replicative suppression of one HCV genotype by another in individual patients. iii) Functional infectious chimeric viruses with polymerase and Env proteins derived from different genotypes have not been reported. iv) Full-length HCV chimeras, engineered with deletions of p7 envelope proteins, are replication deficient and non-infections, indicating intact genotype-specific HCV envelope sequences are essential for proper HCV replication. Specific replacement of p7 of the 1a clone with p7 from an infectious genotype 2a clone was replication defective, suggesting a genotype-specific interaction between the p7 envelope protein and other genomic regions [66]. v) In two independent chimpanzees studies HCV inoculation resulted in persistent infection only in animals developing anti-envelope (E2) antibodies, whereas failure to produce anti-E2 was associated with viral clearance [4,62], intuitively a highly paradoxical result difficult to rationalize unless E2 proteins are important for sustained HCV replication, as we argued previously [45]. vi) Finally, for HCV, specific motifs within the [polymerase] NS5 region of HCV in chronically infected patients predict response to interferon [19,67] an observation that makes little sense unless interferon interacts directly with NS5 [polymerase] motifs, as in-vitro studies suggest [10].

Third, HBV envelope and polymerase protein genes have overlapping open reading frames and significant alterations in envelope and polymerase gene and protein sequences cannot, therefore, occur independently, a genetic nexus again implying an important functional relationship. Mutations in envelope sequences occurring spontaneously [82] following therapy of HBV with lamuvidine and immunoglobulin prophylaxis [6,72] or after vaccine escape [8] are frequently associated with high level viral replication, although replication-deficient mutations are described [47]. These data are generally interpreted to mean polymerase gene mutation(s) cause altered polymerase protein sequence and, hence, abnormal polymerase function. While this is probably partially true if the functionally relevant HBV RNA polymerase is an envelope/polymerase heterodimer (analogous to the p66/p51heterodimer of HIV RT [30]), then an equally valid interpretation is that mutations in envelope genes may change envelope protein conformation and therefore alter normal envelope/polymerase interactions, thus altering processivity and fidelity of the replication complex. This latter interpretation is convincingly supported by data demonstrating that abnormal polymerase function of HBV envelope variants is reversed by co-transfection of Hep G2 cells with clones expressing wild-type envelope sequences [81] and is further supported by clinical studies demonstrating administration of exogenous HBsAg (protein) to patients with chronic HBV dramatically reduced HBV replication [60].

Fourth, studies of the coliphage Qβ demonstrate phage coat proteins bind to genomic RNA [86]to strongly inhibit (association K_ic _≈ 10^7–8 ^M^-1^, inhibition K_i _≈ 10^9 ^M^-1^s^-1^) [79] RNA replication by direct suppression of polymerase activity by envelope proteins [18]. This interaction is dependent on the binding site conformation, but not RNA sequence[86], suggesting interaction avidity will vary as an inverse function of protein sequence divergence from wild type, an intuitive expectation confirmed experimentally [79]. An impressive body of literature documents similar relationships between envelope and polymerase function in swine fever, tobacco mosaic [34], brome mosaic [2] and other RNA viruses. Importantly, studies of the tobacco mosaic virus confirmed this effect to be host-independent and virus-specific inhibition of viral RNA synthesis and to be quite distinct from any interferon effects, intrinsic interference or interference by defective virus [34]. Thus, there exists solid evidence for each necessary component of replicative homeostasis for HCV, HBV and HIV, and other viruses.

### Replicative homeostasis: proposed mechanism

**Figure 4:**
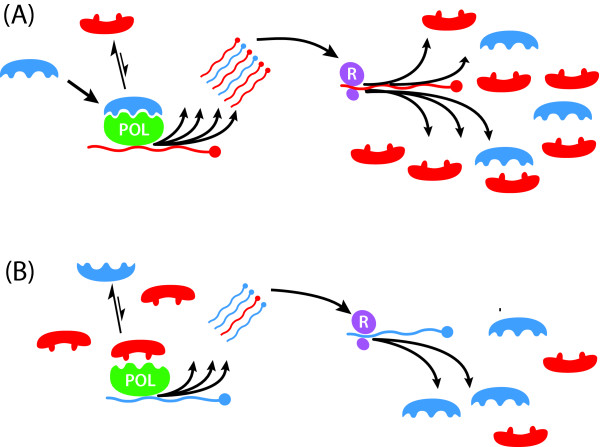
**Mechanism of replicative homeostasis. **At A, relatively high concentrations of Env_Wt _(blue, A) favour high affinity Env:RNA_pol _interactions out-competing variant forms (Env_mt_, red), increasing RNA_pol _processivity but reduced fidelity increasing relative output of variant RNAs. Subsequent ribosomal (R, mauve) translation increases concentrations Env_mt _(red), relative to Env_Wt_, returning the system to equilibrium. Relative excess Env_mt _(B, red) out-compete Env_Wt _(blue) for interactions with RNA_pol_, favouring Env_mt_:RNA_pol_, and blocking Env_Wt_:RNA_pol _interactions. Env_mt_:RNA_pol _complexes relatively decrease RNA_pol_processivity but increase fidelity, increasing output of wild-type RNAs. Subsequent increased translation of Env_Wt _relative to Env_mt _restores the equilibrium.

**Figure 5:**
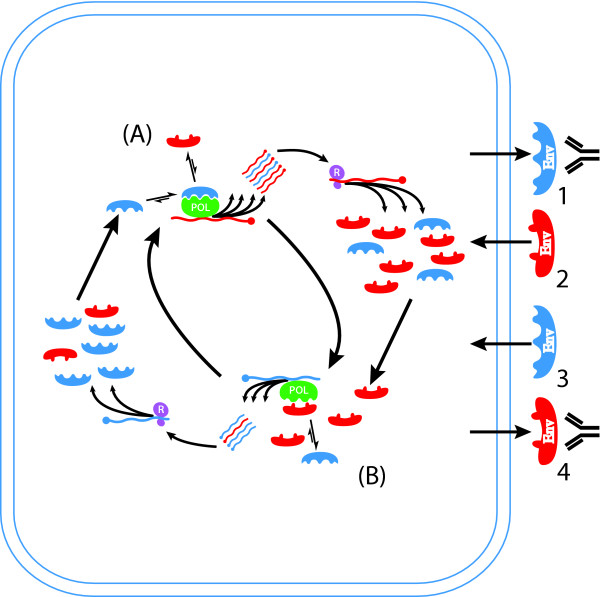
**Conseqences of replicative homeostatic cycles. **Disturbance to intracellular replicative homeostatic cycles. Events increasing intracellular Env_Wt_: Env_mt _ratio (exogenous addition of Env_Wt_, antibody recognition of Env_mt_) will favour Env_Wt_:RNA_pol _interactions, increasing RNA_pol _processivity and reducing fidelity increasing relative output of variant virus. Conversely, events decreasing intracellular Env_Wt_: Env_mt _ratios (exogenous addition of Env_mt_, antibody recognition of Env_Wt_) will favour Env_mt_:RNA_pol _interactions, decreasing RNA_pol _processivity and increasing fidelity, thus reducing replication.

Replicative homeosatsis results from differential interactions of wild-type (Wt) and variant (Mt) envelope proteins on RNA_pol _in a series of feedback epicycles linking RNA_pol _function, RNA replication and protein synthesis (Figure 4, 5). Intracellular accumulation of variant viral proteins causes progressive, direct, inhibition of RNA_pol _and also block Env_Wt_:RNA_pol _interactions that increase replication and mutation. Progressive blockade of RNA_pol _by variant envelope results in a less processive, more faithful, polymerase, increasing the relative output of wild-type envelope RNAs, and, subsequently, translation of wild-type envelope proteins and, hence, an inexorable progression to stable equilibria. Quasispecies stability, and other consequences (including immune escape and low-level basal replication), are inevitable outcomes that result from equilibria reached because of these interactions (Figure 5). We suggest these interactions, and the resulting equilibria, are important therapeutic targets, and the effective ligands – envelope proteins or topologically homologous molecules – implicit within this hypothesis.

Viral polymerases are clearly the effector mechanism – the efferent arm – that determines rate of viral RNA replication and mutation. The afferent arm needs to measure both the rate of viral replication and degree of viral mutation. Intracellular envelope concentrations are a direct function of effective viral replication, while competition between wild-type and variant envelope proteins for interaction with RNA_pol _allows determination of viral mutation rates. Envelope proteins, as opposed to other viral products, are the obvious products to examine for functional variability, and must form part of the afferent arm necessary to "sense" perturbations in the viral equilibrium. While other viral products could be "sensed" to gauge effective viral replication, only functional measurement of envelope protein concentration and topological variability simultaneous measures both the rate of viral replication and envelope functions – properties determined by envelope structure and antigenic diversity – essential for viral survival; immune escape and cell access. Furthermore, envelope and polymerase proteins are typically coded at transcriptionally opposite ends of the viral genome; replication contingent upon a dynamic nexus between envelope and polymerase proteins is, therefore, a functional check of the integrity of the entire viral genome. Importantly, this facet of replicative homeostasis is a direct mechanism of Darwinian selection operating at a molecular level, that ensurs preferential selection and replication of "fit" viral genomes, and maintenance of genotypes (species).

**Figure 6:**
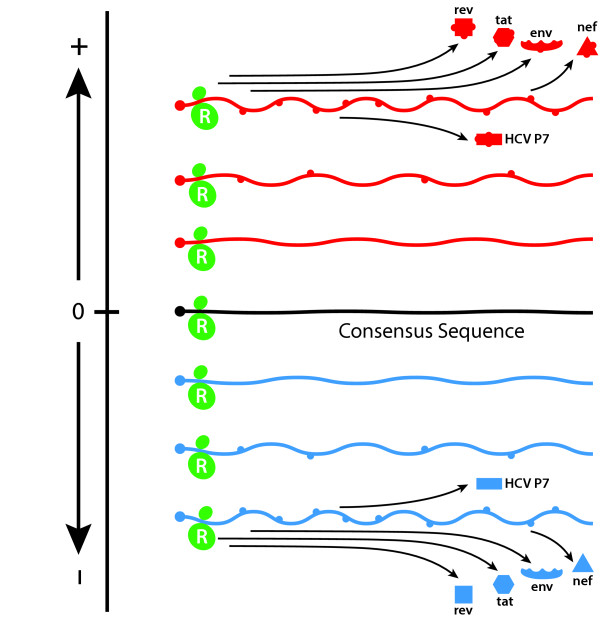
**Phenotypic effects of RNA quasispecies complexity. **Two-dimensional representation of multi-dimensional hyperdense sequence-space that define viral quasispecies; vast RNA /proteins populations progressively divergent from consensus sequence (0). As genetic the distance of RNAs increases from consensus sequence the amino acid sequence, conformation, and functional properties of resulting proteins may also change, potentially resulting in proteins that, despite originating from identical [consensus sequence] genetic domains, have diametrically opposed function. As many accessory proteins (for example, HIV rev, tat, nef and HCV HP7) have open reading frames contiguous with Envelope, sequence changes to Env will also affect accessory protein function.

Viruses, notably HIV, produce many accessory proteins (such as HIV Nef, gag, rev and HBeAg) that affect viral replication and mutation rate. However, these proteins are encoded within envelope open reading frames (ORFs) or are contiguous with them and are likely to alter functionally with any mutation affecting envelope sequences (Figure 6). While these accessory proteins may interact with RNA_pol _(with or without Env) to reset replicative equilibrium (by changing replication rate or mutation frequency or both), stable equilibria will still result providing the sum effect of variant proteins encoded within the envelope ORF is to decrease RNA_pol _processivity (v) and mutation (M_u_) frequency relative to wild-type protein polymerase interactions.

### Testing the hypothesis

This hypothesis is simply tested. Manoeuvres that increase intracellular concentrations of variant envelope proteins or decrease wild-type envelope proteins should inhibit viral replication and reduce mutation rates. Conversely, manoeuvres increasing intracellular [Env_Wt_] or reducing intracellular [Env_mt_] should accelerate viral replication and mutation. In fact, observations relevant to every aspect of this hypothesis have been reported in a variety of systems and circumstances. All outcomes are completely consistent with those predicted by replicative homeostasis. Replicative homeostasis predicts, for example, HCV E2 proteins derived from genotype 1 HCV sequences would reduce HCV replication when administered to patients with heterologous HCV infection (genotypes 2,3 or 4, for example) and studies examining heterologous envelope proteins as direct RNA_pol _inhibitors are underway.

## Discussion

Replicative homeostasis immediately resolves the paradox RNA viral quasispecies stability and explains how these viruses persist and, thereby, cause disease. Replicative homeostasis also explains the initial decline of viral replication, resolving the kinetic paradox, rationalizing the dynamics of chronic viral infection and other enigmatic and unresolved viral behaviours. Most importantly, replicative homeostasis implies a general approach to antiviral therapy.

The equilibria formed by replicative homeostasis are responsive to disturbance of envelope concentrations ensuring viral mutation is neither random nor passive but highly reactive to external influence: Sustained reduction of viral envelope (by immune or other mechanisms) would favour high affinity Env_Wt_: RNA_pol _interactions that, in turn, increase polymerase processivity but reduce fidelity accelerating synthesis of variant viral RNAs and, consequently, increased translation of antigenically diverse proteins, reactively driving quasispecies expansion and generating the extreme antigenic diversity of RNA quasispecies. Alternatively, in the absence of immunological recognition, variant envelope / polymerase interactions predominate, restricting viral replication and mutation, thus maintaining basal output of consensus viral sequences, thus maintaining genotype. Immune escape and maximal cell tropism are inevitable consequences of the potential antigenic diversity generated by RNA replication mediated by the reactive equilibria of replicative homeostasis.

Potential viral antigenic diversity is numerical superior to any immune response; Theoretically, a small envelope protein of 20 amino acids could assume 20^20 ^(about 10^26^) possible conformations, greatly exceeding the ~10^10 ^antibody [80] or CTL receptor conformations either humoral and cellular immune responses can generate. A direct consequence of this mismatch and the stable reactive, equilibria resulting from replicative homeostasis is that once infection is established, the clinical outcome is primarily determined by the viruses' ability to maintain control of the quasispecies, rather than the hosts' response to that quasispecies. This sanguine view is supported by both general clinical experience and by kinetic analysis of chronic viral infection (Figure 2); if host responses are unable to clear virus at 10^5–7 ^viral equivalents / ml they are not likely to be any more effective at 10^8–11 ^eq/ ml.

The varied clinical outcomes of viral infections are explained by replicative homeostasis and its failure: Viral failure to down-regulate replication by RNA_pol _inhibition would cause rapidly progressive or fulminant disease (characterised by massively polyclonal, but ultimately ineffectual, immune responses), while inadequate replication or generation of diversity will result in viral clearance (Figure 3b). Stable, homeostatic replicative equilibria will result in chronic infection with episodic fluctuations in viral replication and host responses (eg ALT; [65]) typical of chronic hepatitis or HIV. The widely varied spectrum and tempo of viral diseases, that for viral hepatitis ranges from asymptomatic healthy chronic carriage to fulminant liver disease and death within days, is far more rationally explained on the basis of a broad spectrum of polymerase properties than highly variable and unpredictable (yet genetically homogeneous) immune responses.

**Figure 7:**
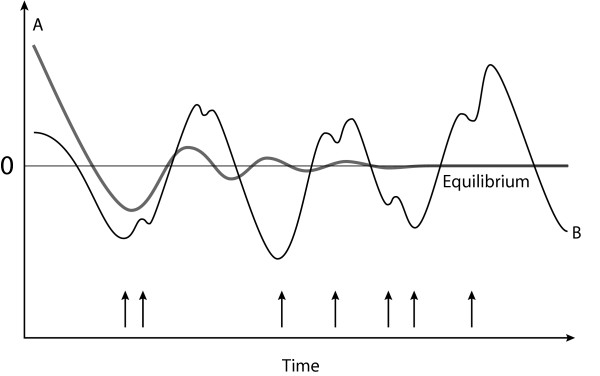
**Homeostatic systems**. In absence of external influence, homeostatic systems (A) progress rapidly to stasis (0) while external perturbations (arrows, e.g. immune recognition of virus) cause pseudo-chaotic fluctuating long-term behaviours in complex systems (B).

Homeostatic systems functioning without external perturbations – such as thermostatically controlled water tanks – progress rapidly to stasis (Figure 7). In tissue culture, viruses – replicating without immune challenge – are unable (and do not need) to generate antigenic diversity by replicative homeostasis, a phenomenon probably responsible for attenuation of virulence of serially passaged virus cultures. By contrast, in dilute viral culture, where viral envelope and polymerase exist in low concentrations, high affinity Env_Wt_/polymerase interactions preferentially occur over lower affinity Env_mt _/polymerase interactions, replicative homeostasis predicts increased viral replication and mutation would occur and this has been confirmed [70]

Perturbations of relative intracellular wild-type and variant envelope concentrations alter RNA_pol_:Env interactions disturbing the replicative equilibria of replicative homeostasis. Antibodies (or CTL) will alter extracellular concentrations of Env proteins, thus changing intracellular envelope concentrations once extracellular /intracellular Env concentrations equilibrate. Therefore, antibodies to heterologous envelope proteins – developing, for example, during immunization against other viruses or heterotypic co-infection – will reduce relative intracellular concentrations of variant envelope, favouring RNA_pol_:Env_Wt _interactions, thus enhancing replication and increasing mutation rates, a prediction confirmed in practice [38,56]. Contrariwise, antibodies to wild-type surface proteins – for example, during administration of anti-HBsAb following liver transplantation for HBV [63] – would reduce viral replication (Figure 6), as seen in practice. Disturbance of viral replicative equlibria by heterologous extracellular antibodies rationally explains antibody-dependent enhancement (ADE) of HIV [23], Dengue [26], Murray Valley encephalitis[84], Ebola [78] Coxsackie [24] and other viruses. Similarly, increased HIV replication and mutation after influenza [38] or tetanus [56] vaccination; reduced HIV replication during measles [50] and Dengue [85] co-infection; clearance of HBV without hepatocyte lysis or evidence of T cell dependent cytotoxicity[25], are also explained by this mechanism. Previously unexplained and problematic viral behaviours and host responses, including long-term non-progression of HIV [7]; persistence of transcriptionally active HBV despite a robust immune response [48]; long-term antigenic oscillations [54]; spontaneous reactivation of HBV[41] (among many other viruses); and hypermutation of HIV, for example, all rationally resolve within this conceptual framework.

There are clear and quite specific therapeutic implications of replicative homeostasis, as well as more general implications. The envelope/polymerase interactions of replicative homeostasis suggested herein are obvious therapeutic targets, and a site of interferon action: Heterologous envelope proteins from different viruses or genotypes of the same virus, or their structural homologues, are likely to inhibit viral replication, as suppression of HIV replication during measles [50] and Dengue [85] co-infection suggests. Interferon is ineffective for HIV and many patients with HBV, and its efficacy in HCV is highly genotype-dependent, strongly implying a direct, virus-specific action unrelated to "immune enhancement", as in-vitro data [10] and clinical kinetic studies imply [52]. Complexing of interferon to RNA_pol _to reduce processivity and increase fidelity would explain both the genotype specificity of interferon action and the kinetics of action and, incidentally, the apparent "immune enhancement" [59] caused by interferon; if interferon reduces RNA_pol _processivity while increasing its fidelity, viral RNAs synthesized will contain fewer mutations causing synthesis of antigenically restricted proteins, thus presenting a more homogeneous target susceptible to immune attack.

Replicative homeostasis may alter perceptions of strategies underpinning the immune responses. It is possible the primary purpose of the initial polymorphic humoral response to viral infection – typically pentameric IgM – is to push viral replication towards equilibria favouring production of homogeneous virus, thus facilitating a concerted and more focussed humoral and/or cytotoxic T cell response; Strong neutralizing IgG antibodies – antiHBsAg, for example – may develop as a consequence of initially restricted viral replication and mutation permitting effective and specific immune recognition, rather than being the proximate cause of it. The temporal profile of HBsAb, that develops well after HBVreplication falls, strongly supports this view. However, once developed, high-affinity neutralizing antibodies against wild-type virus ensure variant envelope proteins remain dominant within cells, thus maximising polymerase inhibition and inhibiting viral replication.

Replicative homeostasis is an adaptation that facilitates stable viral replication in cells and maximises probability of cell-to-cell (and host-to-host) transmission, a prerequisite for viral survival on an evolutionary time scale (Figure 3). A subtle, more primordial, and evolutionary function of envelope/polymerase interactions may explain the origins of replicative homeostasis; Polymerase function contingent upon recognition of, and response to, complex three-dimensional complementarities between polymerase and envelope proteins constitutes a sophisticated encryption technique, effectively "locking" the polymerase, thereby minimises the likelihood any competing RNA (or DNA) molecules are replicated even if correct 5' transcription initiation sequences are present. This is, again, a powerful mechanism of selection, speciation and genotype preservation. As Spiegleman suggested originally [55], in the fierce competition for finite intracellular resources, reproductive strategies that maximise proliferation of "self" genes, while thwarting propagation of "rival" genes, are strongly selected for, and are highly conserved in evolution. The interferons, and other cytokines, are cellular defence mechanisms that long antedate the immune system. If the interferons are functionally homologous to viral envelope proteins, and interact with viral RNApol to reduce processivity and replication to restrict viral replication and antigenic diversity, increasing their susceptibility to immune clearance, it is possible these genes were acquired as result of positive selection of beneficial virus-cell symbiosis occurring early in eukaryotic cellular evolution, a process responsible for retention of other genes [28].

Although proposed specifically to explain RNA viral quasispecies stability, replicative homeostasis is, fundamentally, a mechanism that regulates RNA transcription and modulates protein expression. If proteins (i.e. phenotype) modulate RNA_pol _properties (in a manner contingent on that proteins functionality) and modulate mutations introduced into the RNA templates RNA_pol _synthesises, a subtle form of "quality control" is exerted over protein synthesis [69]. This mechanism accelerates, and directs, adaptation: While introduction of lethal mutations to most RNA genomes may not adversely influence quasispecies, replicative homeostasis ensures any RNA mutations that do arise, and that result in beneficial phenotype(s), will favour replication of that RNA molecule, ensuring that phenotype is retained within the quasispecies. Minor change to polymerase fidelity will profoundly effect a quasispecies; as Haldane demonstrated [27], a reproductive advantage of only 0.1% is sufficient to increase a gene frequency from 0.1% to 50% over a few thousand generations (~1 year for the average patient with HCV) and this effect, therefore, represents a major moulding force in evolution. Thus, replicative homeostasis provides a powerful counterbalance to Muller's ratchet [17] and, by promoting retention and transmission of acquired phenotype, is a Lamarkian mechanism fully consistent with Darwinian principles and operative at a molecular level.

Finally, accessory proteins that alter the processivity and fidelity of both DNA-dependent RNA polymerases [31] and DNA-dependent DNA polymerases [42] to modulate polymerases activity are strongly conserved in evolution, suggesting a critical cellular function. Control of DNA-dependent RNA_pol _transcription by DNA viruses, cellular micro-organisms (e.g. malaria), and eukaryotic cells, subtly modulating cell-surface protein expression, via replicative homeostasis, to mediate immune escape, control cell division and differentiation, or other functions would not be surprising.
